# Cognitive Biases in Chronic Illness and Their Impact on Patients' Commitment

**DOI:** 10.3389/fpsyg.2020.579455

**Published:** 2020-10-28

**Authors:** Lucrezia Savioni, Stefano Triberti

**Affiliations:** ^1^Department of Oncology and Hemato-Oncology, University of Milan, Milan, Italy; ^2^Applied Research Division for Cognitive and Psychological Science, IEO European Institute of Oncology IRCCS, Milan, Italy

**Keywords:** decision making, chronic disease, chronic illness, cognitive bias, patient engagement

## Introduction

Cognitive biases are constructs based on erroneous or deformed perceptions which produce systematically distorted representations with respect to some aspects of the objective reality, such as prejudices (Haselton et al., 2005). Biases impact everyday life because they affect decisions and behaviors. For example, one may persist in an unhealthy behavior (e.g., smoking) because he selectively overestimates evidence that feeds up a pre-existing conviction (e.g., “smoking boosts my concentration”) (Masiero et al., 2019): this is known as confirmation bias (Hernandez and Preston, 2013).

While some biases appear inherent to human cognition, others are situation–specific. Several studies have shown that there are cognitive biases typical of people who live with a chronic illness and continually attend to health management (Lichtenthal et al., 2017). These biases influence information processing about the disease and consequently decision making (DM), impacting the health and quality of life (Khatibi et al., 2014). The objectives of the present contribution are to synthetize information on biases in chronic illness and to highlight the possible effect of biases on health management. The last sections will explore how biases could influence not only the information processing, but also the motivation and agency within the patients' healthcare journey.

## Cognitive Biases in Chronic Illness

DM in chronic illness is complex because patients find themselves in a state of uncertainty (Reyna et al., 2015), and have to take life-relevant decisions in an emotionally-charged situation (Szekely and Miu, 2015; Mazzocco et al., 2019). People are averse to the unknown and risk (Tversky and Kahneman, 1986), and this may lead them to choose suboptimal treatments because they are perceived as less risky. For example, a patient may decide to refuse a treatment as it involves unlikely yet feared risks, this way failing to consider the benefits (Fraenkel et al., 2012; Pravettoni et al., 2016). The biases most frequently highlighted in the literature on chronic illness are attentional (Bar-Haim et al., 2007; Chan et al., 2011), interpretation (Ouimet et al., 2009; Lichtenthal et al., 2017), and recall biases (Karimi et al., 2016). Attentional bias is defined by Schoth et al. (2012) as the selective attention to specific information, failing to consider the alternatives because of the interference of pre-existing sensitivity. Interpretation bias is the patients' tendency to interpret an ambiguous information in an illness–related fashion and to catastrophize (Crombez et al., 2013; Khatibi et al., 2015). Recall bias consists in distortions in the accuracy of the recollections retrieved (“recalled”) about events or experiences from the past (Last, 2000).

These biases have, in common, the tendency to prioritize information connected to the disease/illness experience, at any level of information processing and DM. For example, individuals tend to selectively focus on threat or pain–related words or pictures (Bar-Haim et al., 2007; Crombez et al., 2013). Attention to threatening stimuli and illness–related interpretation can lead to biased decisions in terms of treatment and lifestyle: subjects with chronic pain will tend to focus on pain–related information and consequent preoccupation (Bar-Haim et al., 2007; Hakamata et al., 2010; Schoth et al., 2012), this way preferring healthcare options that are less likely to cause pain, independently of their overall effectiveness or value. Similarly, they would avoid certain activities they feel potentially pain–inducing, with the consequence of social isolation and reduced social support (McCracken, 2008; Schoth et al., 2012). Negative interpretation of information influenced by interpretation bias could promote a greater pessimism about the potential control of a disease and, therefore, lower the implementation of control behaviors which are considered ineffective (Miles et al., 2009; Everaert et al., 2017).

Studies in psycho-oncology have shown that biases play a role in the fear of recurrence (FOR) (Miles et al., 2009; DiBonaventura et al., 2010). The fear that cancer may return, an important aspect to monitor in cancer survivors (Marzorati et al., 2017; Tsay et al., 2020), features a cognitive component related to the survivor's difficulty in processing disease–related information, thus, reducing the understanding of pathology and treatment. Patients with FOR tend to focus on the negative aspects within the doctors' explanation (Wenzel and Lystad, 2005; Davey et al., 2006; Han et al., 2006). Possible consequences entail detriment to the patient-doctor alliance (Ha and Longnecker, 2010), patient's inability to take into account all aspects of medical information to take good decisions (Kee et al., 2018), and, in the long run, the tendency to resort to options alternative to traditional medicine patients feel reassuring (Dobrina et al., 2020).

For what regards recall bias, people with past experience of pain or suffering create memory traces that distort the memory of a stimuli associated with those sensations (Karimi et al., 2016). Some studies on patients with chronic pain have shown propensity to recall pain–related information (Pincus and Morley, 2001; Rusu et al., 2012). Studies have demonstrated a recall bias for somatic symptoms showing a retrospective overestimation of symptom severity (Broderick et al., 2008; Walentynowicz et al., 2015). Lindberg et al. (2017) showed that breast cancer survivors' perception of past quality of life is significantly worse than it actually was (physical and cognitive functioning, fatigue, and pain). Patients with depression and pain recalled negative health–related information to a greater extent than the non-depressed controls and patients with depression or pain only, showing that the recall bias is exacerbated both bythe psychopathological and physical condition (Rusu et al., 2012). While there is less information on the direct influence of recall bias on health management, the propensity to recall negative information may affect the patients' self-efficacy or their belief to be able to manage their own health, in that memory of successful management (“mastery”) is crucial to the maintenance of motivation (Hiltunen et al., 2005). In other words, it would hinder the perception of an effective self-agency which is necessary to implement healthy behaviors and treatment adherence, especially when it requests effort on the patient's side.

### Biases in Self-Perception

The tendency to focus on a threatening stimuli may affect a chronic patient's cognition on a deep level. According to literature, this tendency may be rooted in self-perception. Self-perception is defined as the “cognitive generalizations about the self, derived from past experience, which organize and guide the processing of self-relevant information contained in the individual's social experience” (Markus, 1977, p. 64). Self-perception may be distorted (Alloy et al., 1988; Walfish et al., 2012). Chronic patients may develop self-perception focused on illness–related memories, such as viewing themselves as “sick” or “injured.” Indeed, chronic disease implicates years of experience, adaptation to a disease of varying severity, making this information highly accessible. On one hand, self–related biases influence distorted tendencies in information processing such as those outlined above (attentional, interpretation, and recall biases) (Derry and Kuiper, 1981; Clemmey and Nicassio, 1997; Guzman and Nicassio, 2003). On the other hand, illness–related self-representation could be directly associated with mental health outcomes, such as anxiety and depression (Triberti et al., 2019), especially when the current (“actual”) self is perceived inconsistent with other coexisting self-representations (e.g., the “ideal self” or the person one would like to be), a phenomenon known as “self-discrepancy” (Higgins, 1987, 1989). This result emerged for example in a research where oncological patients were asked to create digital avatars representing their multiple facets of the self (Triberti et al., 2019), as well as in qualitative and quantitative research focused on the chronic patients' self-perception (Clemmey and Nicassio, 1997; Bailly et al., 2015; Michaelis et al., 2019). Recent reviews highlight that self-discrepancy represents a contributory factor in psychiatric disorders (Mason et al., 2019) and negatively affects the patients' quality of life (Kwok et al., 2016).

### Social Biases

Full consideration of biases within the chronic illness context requires taking into consideration those related to social cognition. DM rarely occurs in isolation. Indeed, the decisions in a chronic illness are often influenced by others (Ellickson et al., 2005; Germar et al., 2014). Others' influence on decisions can often lead to a wrong evaluation of the choices with a tendency to take a greater risk (Gardner and Steinberg, 2005; Muchnik et al., 2013). Social biases can occur within the social context. Several studies have dealt with the study of group psychology (Bar-Tal, 2012; Hogg, 2012; Thibaut, 2017); for example, the classic experiment by Asch (1951) showed that a subject will tend to conform his opinion, even when clearly untrue, to that of the other members of the group he feels part of because of social pressure. Groups may exert an influence on the cognitive processes and decisions just by a conformity effect. Certainly, such classic experiments may be criticized today, for example, because they rely on abstract tasks and artificial settings and have a low ecological validity (Arjoon, 2008). Yet, it is well-known that groups belonging could promote biases in reasoning. Chronic patients are influenced by caregivers, family, and close friends, who often have different preferences regarding the treatment (Laryionava et al., 2018). Furthermore, health and medicine have now become an increasingly shared context online; patients have access to information that is not always reliable and evidence–based, and they may join groups more easily, often with the aim to share experiences, receive advice, and empathic support. The well-known example of anti-vaccine groups and related studies (Jolley and Douglas, 2014) show that the exposure to conspiracy theories within groups may sensitively affect the patients' health decisions. Even in the case of chronic patients, a social bias can, therefore, lead the patients to change their attitudes and opinions in favor of those shared by relevant groups.

## The Influence of Biases on the Patients' Decision Making

Biases can influence the DM process in chronic illness (Gorini and Pravettoni, 2011; Lucchiari and Pravettoni, 2013). Some cognitive biases in chronic illness could enhance attention to and the salience of symptoms which tend to be perceived as uncontrollable and incurable (Moss-Morris and Petrie, 2003), so that they negatively influence the patients' decisions regarding treatment and health management. Furthermore, patients affected by biases in self-perception may find themselves in a situation of perceived helplessness and self-derogation, which affects their ability to manage their own health and possibly augments the risk of mental health issues, such as anxiety. Psychologically vulnerable chronic patients could also refer to others and groups to make health decisions, which is a risky strategy especially when unprofessional opinions are involved.

**Figure 1:**
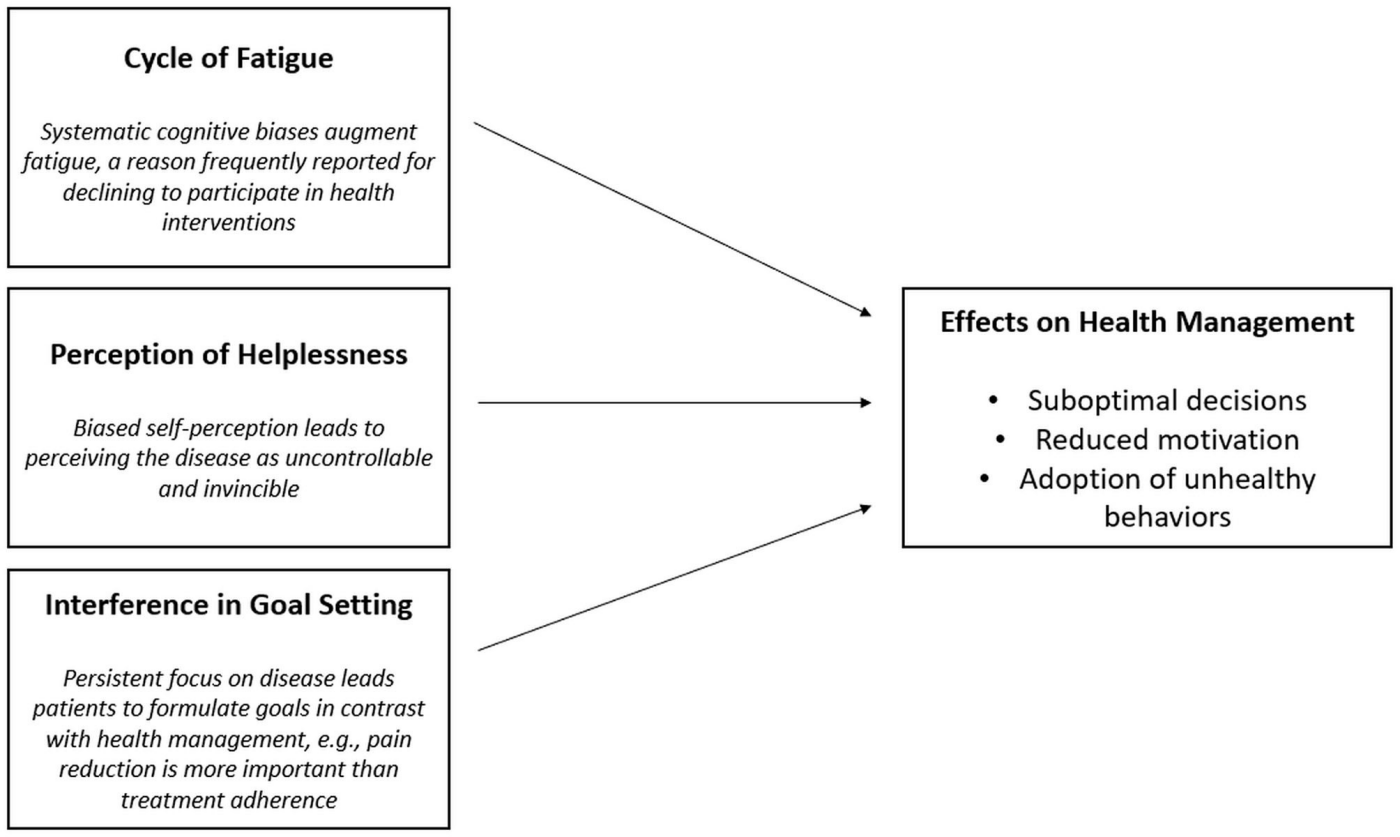
Three main processes that influence patients' health management.

It is possible that biases in chronic illness could influence DM and the formation of effective motivation to engage in healthy behaviors. Many psychological interventions are conducted to help patients manage their own health, as well as to recover a sense of authority and control over their life, this way addressing the biases' effects (Kondylakis et al., 2017). However, the patients' decision to take part in such interventions could be influenced by biases as well. Among the multiple possible mechanisms, we hypothesize that this happens because of three main processes (Figure 1). The first involves fatigue as psychological process directly related to biases. Recent studies have underlined that a reason to decline participating in a psychological intervention or resorting to psychological support is feeling tired or weak (Bernard-Davila et al., 2015; Aycinena et al., 2017). Indeed, it exists as a reciprocal interaction between the systematic biases and perception of fatigue: on the one hand, fatigue (physical and cognitive) leads to a careless information processing which augments the likelihood of biased reasoning (Boksem and Tops, 2008; Howard et al., 2015); on the other hand, symptom focusing and the way chronic patients interpret disease–related information are demonstrated to augment their perception of fatigue (Wiborg et al., 2011; Hughes et al., 2016).

Another relevant process regards the perception of helplessness as a self-perception component. Helplessness leads subjects to perceive symptoms like chronic pain as uncontrollable, unpredictable, and immutable, and to generalize these to daily functioning (Abramson et al., 1978; Evers et al., 2001). Along with passive coping (activity avoidance and persistent worrying), this contributes to perceiving the disease as uncontrollable and invincible, reducing self-efficacy, and the motivation to react to it (Samwel et al., 2006; Verhoof et al., 2014).

Finally, it is possible that the influence of systematic biases is pervasive to the point that it influences motivation formation. While motivation is often conceptualized as a dynamic force or pull (e.g., drive, instinct, intention), it could be structured as the declarative, explicit course of actions and outcomes to achieve, namely *objectives* or *goals* (Ryan, 2012; Triberti and Riva, 2016). Goal setting is a fundamental component of any care plan (Vaughn et al., 2016). Goal setting allows patients to identify the short- and long-term objectives to achieve, taking into account the patient's needs and lifestyle (Wade, 2009; Levack et al., 2015; Smit et al., 2019). Biases and, in particular, the tendency to focus on the negative factors may lead the patients to formulate goals to avoid the negative symptoms (e.g., pain), instead of pursuing the long-term personal growth objectives (e.g., “I will not participate in the intervention because it's tiring: I just need to rest”).

On this basis, it is possible that systematic cognitive biases in chronic illness do not only influence the treatment decisions but also the motivation to resort to interventions that could help in reduce their detrimental effects. In other words, the repeated influence of the cognitive biases may be associated with a “vicious circle” that reduces the patients' motivation to recognize and address the same mental health issues that influence their DM.

## Conclusion

The present contribution explored the ways biases could influence the motivation and agency within the patients' healthcare journey. By considering of chronic illness biases, we hypothesized that DM and motivation are directly altered, leading to a reduced patient engagement in their own healthcare. The strength of this hypothesis lies in the possibility to test it by quantitative research focused on the prevalence of specific biases in patient populations characterized by a low engagement and/or by the tendency to decline participation in health interventions. On the other hand, its weakness lies in the possibly reciprocal interaction between the biases and engagement: patients may incur in frequent biased cognition exactly because they are not adequately supported in their care process. Furthermore, the three mechanisms hypothesized here do not exhaust all the possible influences of biases so that future research should provide evidence to build a more complete model of their effects on the patients' decision making. This would allow the practitioners to understand how to address dysfunctional cognition to improve the accessibility and effectiveness of health engagement interventions.

## Author Contributions

LS conceived the ideas presented in the article and wrote the first draft. ST contributed with the discussion on the ideas presented and supervised the writing. Both authors contributed equally to the revision.

## Conflict of Interest

The authors declare that the research was conducted in the absence of any commercial or financial relationships that could be construed as a potential conflict of interest.
